# Chemokine family significance and prognostic value in colorectal cancer

**DOI:** 10.3389/fimmu.2024.1404768

**Published:** 2025-01-06

**Authors:** Yi Ding, Yinnan Chen, Siyun Xie, Quanpeng Qiu, Xiaolong Guo, Yun Feng, Hongxia Li, Fang Zhu, Yaping Liu

**Affiliations:** ^1^ Department of Gastroenterology, The First Affiliated Hospital of Xi’an Jiaotong University, Xi’an, Shaanxi, China; ^2^ Department of General Surgery, The First Affiliated Hospital of Xi’an Jiaotong University, Xi’an, Shaanxi, China; ^3^ Department of High Talent, The First Affiliated Hospital of Xi’an Jiaotong University, Xi’an, Shaanxi, China; ^4^ Center for Gut Microbiome Research, Med-X Institute, The First Affiliated Hospital of Xi’an Jiaotong University, Xi’an, Shaanxi, China; ^5^ Hubei Province Key Laboratory of Precision Radiation Oncology, Wuhan, China; ^6^ Information Science and Technology, Northwest University, Xi’an, Shaanxi, China; ^7^ Clinical Medical Research Center for Digestive Diseases of Shaanxi Province (Oncology), The First Affiliated Hospital of Xi’an Jiaotong University, Xi’an, Shaanxi, China; ^8^ Cancer Center, Union Hospital, Tongji Medical College, Huazhong University of Science and Technology, Wuhan, China

**Keywords:** chemokines, colorectal cancer, prognostic models, immune microenvironment, immune infiltration

## Abstract

**Background:**

Colorectal cancer (CRC) poses a substantial global health concern, exhibits inconspicuous early symptoms, and is typically diagnosed at advanced stages leading to unfavorable outcomes. The intricate tumor microenvironment plays a crucial role in CRC development and progression, where chemokines contribute significantly. These chemokines exhibit widespread expression within tumor cells, facilitating immune cell infiltration, angiogenesis, and the establishment of distant metastases. The dysregulation of various chemokines in the context of CRC has emerged as a pivotal factor in the disease's pathogenesis.

**Methods:**

To explore the relationship between chemokine gene expression and CRC patient survival, as well as to clarify their biological roles,We conducted RNA-sequencing (RNA-seq) analysis on a cohort of 88 CRC patients with tumor samples, thereby enabling a detailed exploration of chemokine involvement in CRC. This study was rigorously augmented using comprehensive datasets from The Cancer Genome Atlas (TCGA), ensuring a robust analysis of gene expression patterns associated with clinical outcomes.

**Results:**

Through data analysis, we identified key genes from the chemokine family thought pertinent to CRC outcomes. Consequently, we constructed a novel prognostic model based on the risk score derived from these chemokine expressions. Validation against clinical metadata, executed through immunohistochemistry analysis, affirmed the relevance and accuracy of our model in predicting patient survival.

**Conclusion:**

Our findings illuminate the critical role of chemokines in shaping the immune microenvironment of CRC, thereby highlighting potential therapeutic targets for future treatment strategies. Our new prognostic model could provide important information for the development of targeted therapies for CRC, enhancing personalized treatment approaches andultimately improving survival for CRC patients.

## 1 Introduction

Colorectal cancer (CRC) is the third most common cancer and the second leading cause of cancer-related deaths worldwide, causing nearly 700,000 deaths annually (1). Early manifestations of CRC are often difficult to detect, making timely diagnosis difficult. As the disease progresses, symptoms such as blood in the stool and abdominal pain may occur. However, in advanced stages, the prognosis deteriorates significantly. At present, the cornerstone of CRC treatment is the combination of surgery and radiotherapy, which is the most effective form of treatment. However, even after meticulous surgical resection, a significant number of patients have to face the frustrating reality that 40 to 50 percent of them are left with recurrent disease or metastatic invasion (2). In addition to the complexity of treatment, a significant proportion of CRC patients develop resistance to chemotherapy, worsening the already bleak outlook for survival (3). It is therefore imperative to develop more effective therapeutic strategies to combat CRC.

Currently, a large number of studies have found that chemokines regulate CRC development, which may be related to the fact that chemokines influence the generation and type of immune cells in the tumor microenvironment (4–6). Chemokines are small secreted proteins that bind to G protein-coupled receptors (7). They are mainly involved in regulating physiological processes such as organ development, immune surveillance, host defense and tissue renewal and regeneration. chemokines play an essential role in inflammatory and immunological processes (8). chemokines are classified into four main subfamilies: CC, CXC, XC, and CX3C (9). In the intricate tumor microenvironment (TME), chemokines directly affect tumor cells and endothelial cells and regulate important processes such as tumor cell proliferation, angiogenesis, cancer stem cell properties, invasiveness, and metastasis (10). It has been established that CXCL11 has been associated with enhanced antitumor immunity and a favorable prognosis in CRC (11). With notable achievements, tumor immunotherapy focused on the chemokine system has recently been introduced (12, 13).Although chemokine and chemokine-receptor-based therapies for CRC have yet to be integrated into clinical practice, the CXCR4 inhibitor LY2510924 has exhibited a favorable clinical safety profile and tolerability, demonstrating a 20% overall response rate in phase I clinical trials (4). These advancements have propelled the acknowledgment of chemokines and their receptors as promising targets for cancer immunotherapy. The potential of chemokines in cancer immunotherapy holds promise; however, their specific roles in the context of CRC remain largely unexplored. Consequently, a comprehensive understanding of the role and mechanisms of the chemokine system in colorectal carcinogenesis biology becomes imperative. Our ongoing research efforts aim to refine the prognostic assessment of CRC through the construction of prognostic models by chemokines, and ultimately advance CRC-targeted therapeutic strategies by elucidating the biological functions and properties of chemokines.

## 2 Method

### 2.1 Patient data sets

Eighty-eight patient samples were collected from the First Affiliated Hospital of Xi’an Jiaotong University, which complied with hospital ethical requirements. COADREAD data was retrieved from TCGA. mRNA expression data (698 samples) and clinical information were simultaneously extracted from the TCGA database (https://cancergenome.nih.gov). This work strictly adheres to both TCGA publication requirements.

### 2.2 Chemokine expression in CRC

The “limma” and “pheatmap” R packages were used to analyze the expression of differentially expressed chemokines in tumor and normal tissues. P<0.05 indicated statistical significance. The correlation of chemokine expression was evaluated with Spearman correlation analysis. It was determined that P<0.05 was statistically significant.

### 2.3 Functional enrichment analysis

The DEG threshold for functional enrichment analysis is defined as an adjusted P<0.05. gene ontology (GO) including biological process (BP), cellular component (CC) and molecular function (MF), and Kyoto Encyclopedia of Genes and Genomes (KEGG) analysis using the clusterProfiler package (version 3.14.3 version) for enrichment analysis; org.Hs.eg.db package (version 3.10.0) for ID conversion.

### 2.4 Correlation of immune cell infiltration

The Tumor Immune Evaluation Resource (TIMER) database (14) was used to analyze the correlation between differentially expressed chemokines and tumor-infiltrating immune cells (B cells, CD4+ T cells, CD8+ T cells, neutrophils, macrophages, and dendritic cells). A timer algorithm was used to estimate the abundance of the six immune infiltrates in R(4.1.0).

### 2.5 IHC

Immunohistochemical images of XCR1 and CCR10 expression in CRC tissues and normal tissues from Human Protein Atlas (https://www.proteinatlas.org/). We performed IHC on normal and CRC tissues from 10 patients. In this experiment, formalin-fixed and paraffin-embedded tissue sections were deparaffinized and boiled in sodium citrate buffer (pH 6.0) for 45 minutes using a microwave tissue processor. Sections were treated overnight at 4°C with mouse anti-human CCR10 (polyclonal antibody, 22071-1-AP, Proteintech) antibody with rabbit anti-human XCR1 (polyclonal antibody, DF9046, Affinity Biosciences). In addition, quantitative analysis of IHC-positive expression was performed using ImageJ software.

### 2.6 Quantitative reverse transcription PCR

Total RNA was extracted from tissue homogenates using Trizol reagent (Solarbio, Beijing, China) according to standard protocols. the same amount of RNA (1 μg) was reverse-transcribed using StarScript II Reverse Transcription Kit (Genstar, Beijing, China). Complementary DNA (cDNA) was analyzed by qPCR using 2× RealStar Fast SYBR qPCR Mix reagent (Genstar, Beijing, China) at a final dilution of 1:5. The primer sequences were as follows:

CCR10: 5′-GCAAACGCAAGGATGTCGC-3′, 5′-CGTAGAGAACGGGGATTGAGGC-3′;

XCR1: 5′-ATGGAGTCCTCAGGCAACC-3′,5′-CGAGGGTAGCAAAGACCCA-3′;

CXCL13: 5′-GCTTGAGGTGTGTAGATGTGTCC-3′,5′-CCCACGGGGGCAAGATTTGAA-3′;

CXCR6: 5′-GCACACACTGGGAATACTATGC-3′,5′-CCCTCAGGTATGCGATGGC-3′.

### 2.7 Modeling and testing chemokines models to predict prognosis in CRC patients

Kaplan-Meier analysis was used to obtain chemokines with P<0.05, which were associated with the prognosis of CRC and had prognostic value. Then, multifactorial COX regression analysis was performed to screen out the meaningful chemokines among them to construct CRC-related prognostic markers, and risk scores were calculated. Patients were grouped according to survival and death, and a heat map of gene expression under the survival profile was constructed based on prognostic features combined with risk scores. All CRC patients were categorized into high and low groups based on the median risk score. K-M survival curves were plotted, and the difference in OS between the two groups was evaluated to be statistically significant by the log-rank test. In addition, we plotted ROC curves using the “Survival ROC” program in R(4.1.0) to verify the accuracy of the model. Principal component analysis was performed based on the “prcomp” program package in R (4.1.0). Univariate and multivariate analyses were performed using the COX regression method to verify whether the risk score could be used as an independent prognostic factor for CRC. The likelihood of the model predicting the prognosis of CRC patients was examined by nomograms.

### 2.8 Statistical analysis

Kaplan-Meier analysis was employed to identify chemokines exhibiting statistical significance (P<0.05) about the prognosis of CRC, thereby demonstrating prognostic relevance. Subsequently, multifactorial COX regression analysis was conducted to discern meaningful chemokines from the identified candidates and to establish CRC-specific prognostic markers. Risk scores were computed accordingly. Patients were stratified into survival and deceased cohorts, and a heatmap illustrating gene expression profiles corresponding to survival outcomes was generated based on prognostic attributes and risk scores. Subsequently, all CRC patients were dichotomized into high and low-risk groups based on the median risk score. Kaplan-Meier survival curves were constructed, and the divergence in overall survival (OS) between the two groups was assessed for statistical significance using the log-rank test. Additionally, Receiver Operating Characteristic (ROC) curves were generated utilizing the “Survival ROC” program in R (version 4.1.0) to validate the model’s accuracy. Principal component analysis (PCA) was executed utilizing the “prcomp” package in R (version 4.1.0). Univariate and multivariate analyses were performed via COX regression to ascertain whether the risk score could serve as an independent prognostic determinant for CRC. Nomograms were utilized to assess the model’s predictive capability concerning the prognosis of CRC patients.

## 3 Result

### 3.1 Variation of chemokines family in CRC

**Figure 1:**
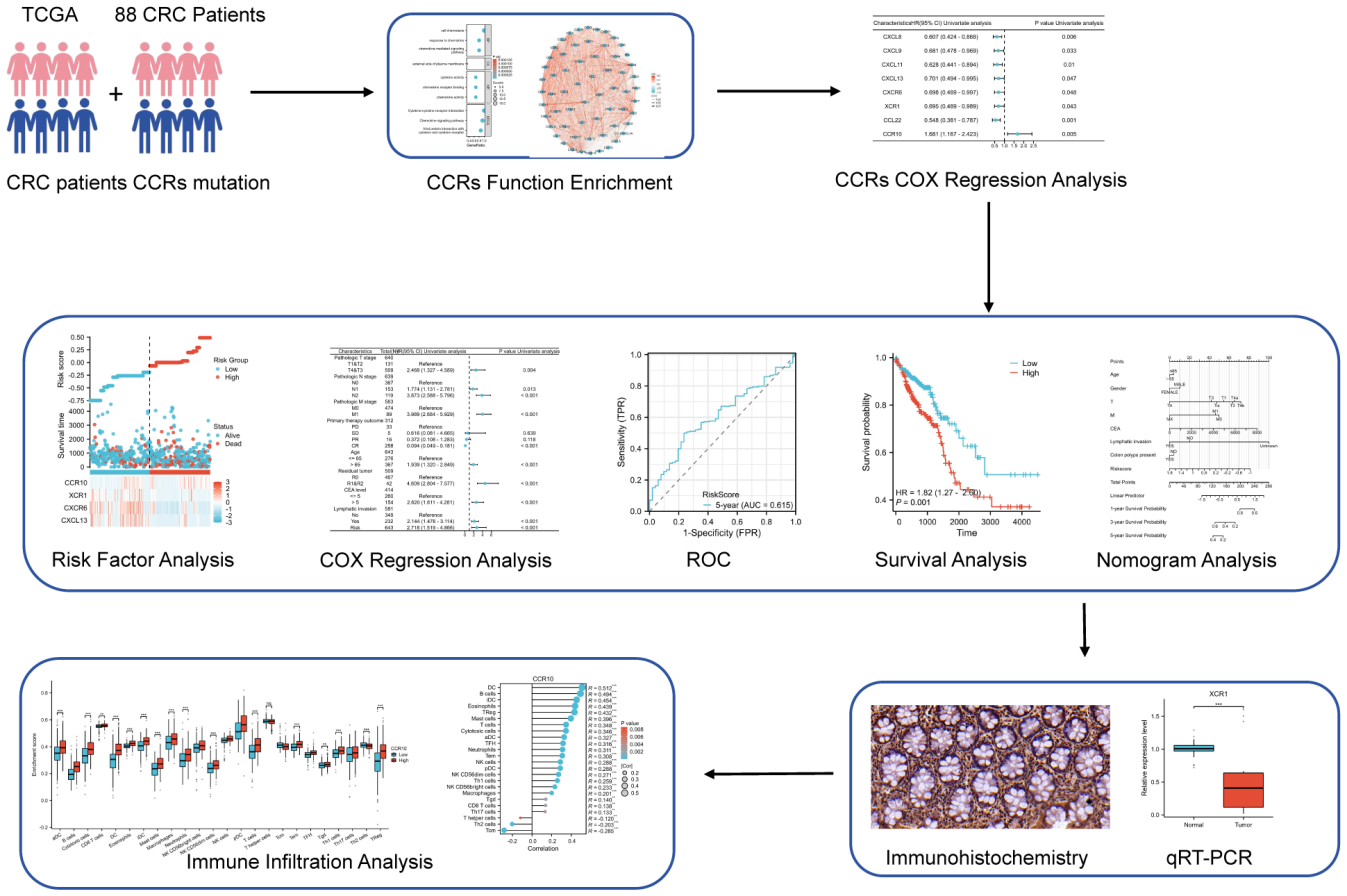
The flow chart of the current study.

**Figure 2:**
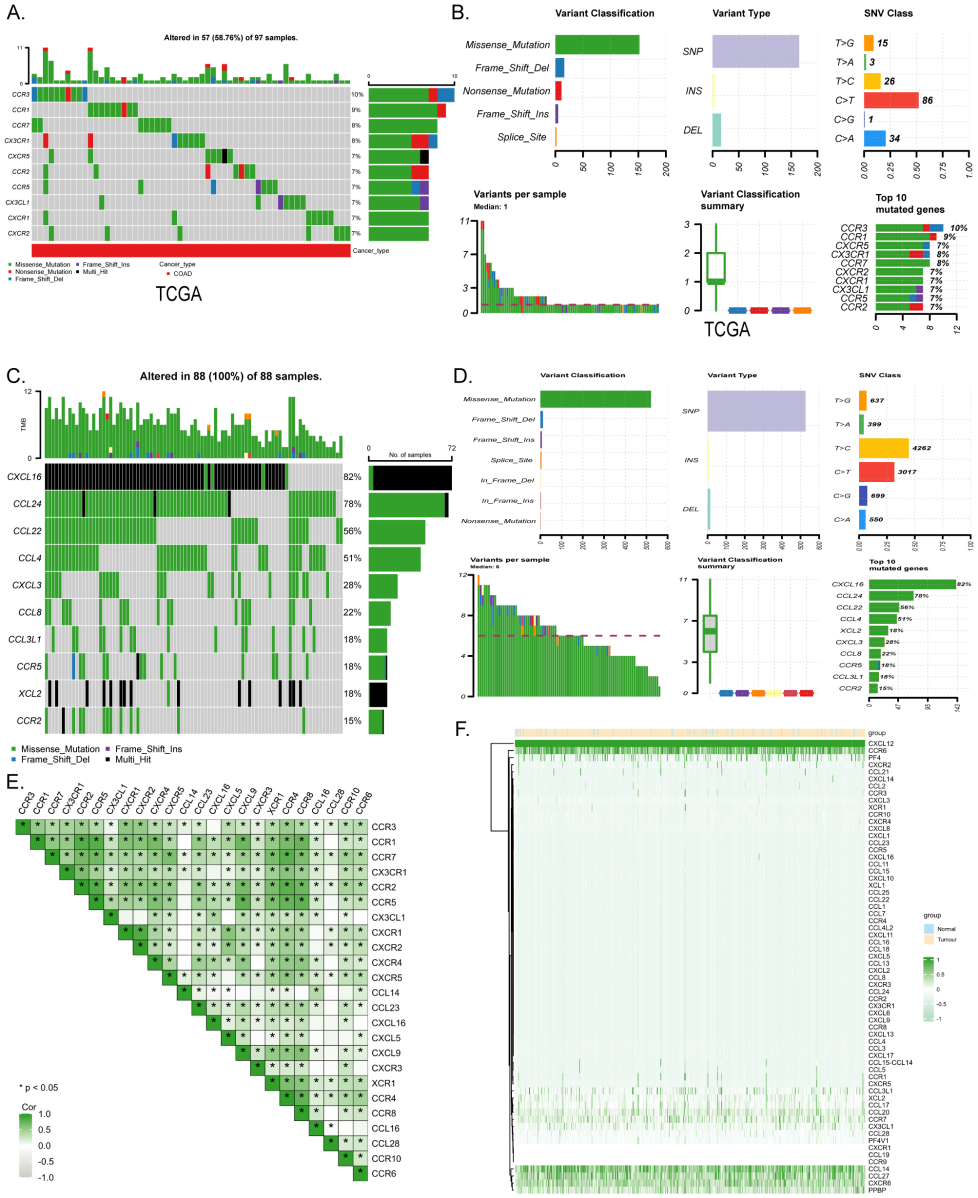
Mutation and expression analysis of chemokines in CRC. **(A)** Top 10 mutated chemokines in COADREAD samples from TCGA database. **(B)** chemokine mutation profile in TCGA-COADREAD dataset. **(C)** Mutation profiles of the top 10 chemokines from the clinically collected independent cohort. **(D)** chemokine mutation profiles from the clinically collected independent cohort. **(E)** Expression correlation of 24 significantly expressed chemokine. **(F)** Heatmap of chemokines expression in normal and CRC tissues in the TCGA database. p < 0.05 *.r

The research flow chart depicts the study design (**Figure 1**). A prognostic model and immune infiltration analysis of colorectal cancer patients was developed by combining TCGA with independent data from 88 colorectal cancer patients. and the model was validated through self-collected clinical cohort information, immunohistochemistry, and qRT-PCR (**Table 1**). We analyzed the frequency of copy number variants and somatic mutations within 64 chemokines obtained from CRC specimens (15). Among the 97 samples of colorectal cancer, mutations were found in 57 samples, with a mutation frequency of 58.76%. chemokine3, chemokine1, chemokine7, CX3CR1, CXCR5, chemokine2, chemokine5, CX3CL1, CXCR1, and CXCR2 emerged as the ten most commonly mutated genes. Among these, chemokine3 and chemokine1 exhibited the highest mutation rates, approximately 10% each (**Figure 2A**). The predominant forms of mutations observed were missense mutations, shift deletions, and nonsense mutations, and has the highest number of C>T SNV classes (**Figure 2B**). Subsequently, data from 88 CRC patients at the First Affiliated Hospital of Xi’an Jiaotong University were examined, focusing on mutations within the chemokines family. Noteworthy genes frequently affected by mutations included CXCL16, CCL24, CCL22, CCL4, CXCL3, CCL8, CCL3L1, chemokine5, XCL2, and chemokine2 (**Figure 2C**). Consistently, missense mutations, shift deletions, shift insertion mutations predominated, SNV Class has the most T>C and the second most C>T, and aligned well with TCGA data (**Figure 2D**). Furthermore, Spearman correlation analysis was conducted to evaluate co-expression correlations among the 24 most significantly differentially expressed chemokine genes out of the 64 studied in CRC. Positive correlations in gene expression were noted in CRC, with CXCL9 and CXCL10 exhibiting the strongest positive correlation (R=0.91) (**Figure 2E**). Despite these findings, genetic variation remains a significant factor influencing chemokine expression. To further explore this aspect, the expression profiles of 64 chemokines were assessed across 698 tumors and normal tissues sourced from the TCGA database. From the heatmap, we can see that the expression levels of XCR2 and CX3CL1, which have high genetic variability, are also relatively high (**Figure 2F**).

**Table 1:** Basic patient information.

Characteristics	overall
N stage, n (%)	
N0	50 (56.8%)
N1	18 (20.5%)
N2	20 (22.7%)
M stage, n (%)	
M0	81 (92%)
M1	6 (6.8%)
Mx	1 (1.1%)
Age, mean ± SD	63.841 ± 9.5879
Gender, n (%)	
Man	48 (54.5%)
Woman	40 (45.5%)
Height, mean ± SD	166 ± 8.5501
Weight, mean ± SD	65.511 ± 12.066

### 3.2 Analysis of expression differences and functional enrichment

**Figure 3:**
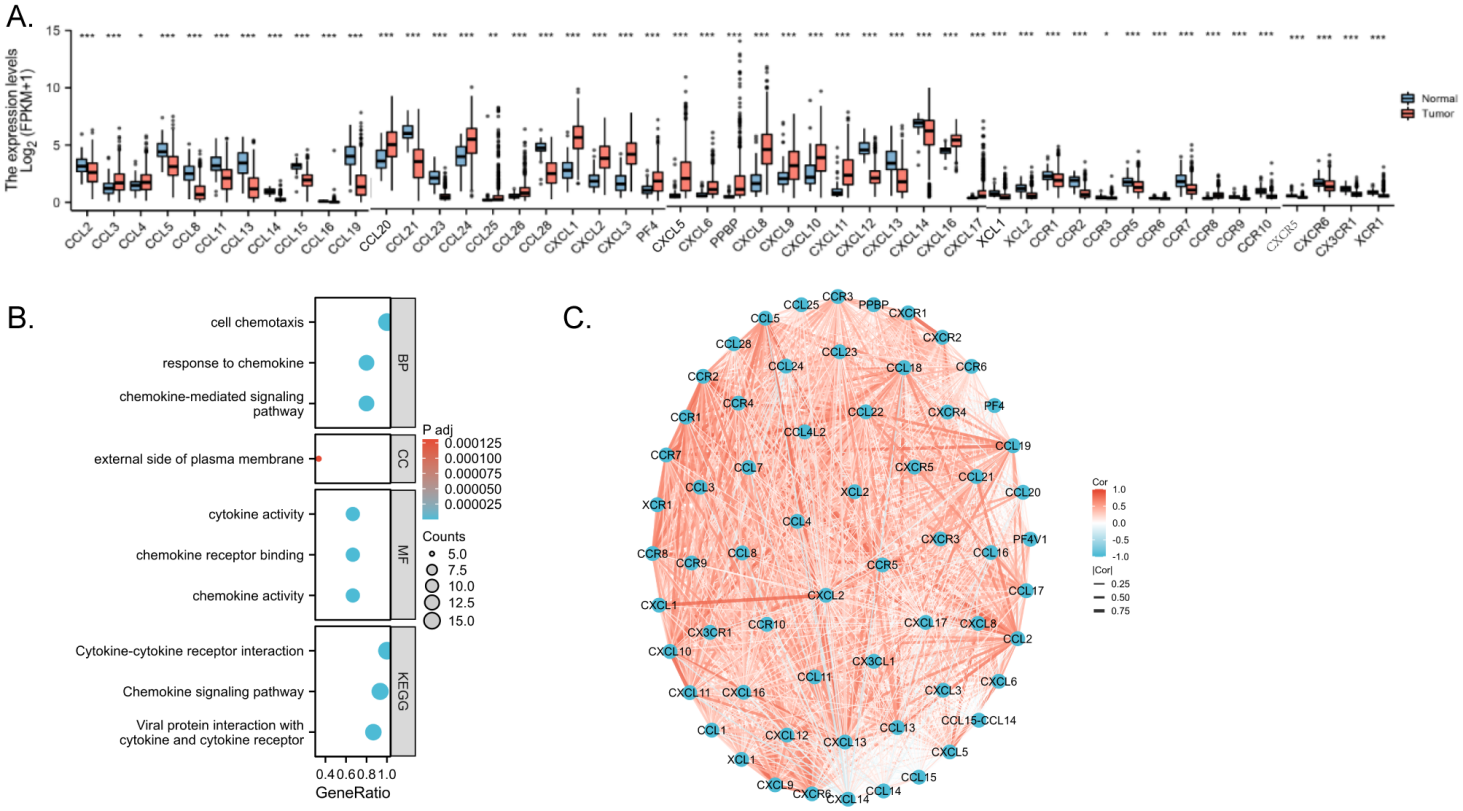
Enrichment analyses and PPI network. **(A)** Correlation between chemokines. Red represents positive correlations and blue represents negative correlations. Spearman correlation analysis was used for correlation analysis. **(B)** GO/KEGG enrichment analysis **(C)** Network of intermolecular interactions linking chemokines. p < 0.05 *, p < 0.01 ** and p < 0.001 ***.

Integrated analysis of the TCGA database unveiled differential expression of chemokines between CRC and normal tissues, from which the median expression of chemokines was selected for comparison, with 48 genes within the chemokines family exhibiting statistically significant differences (P<0.05) (**Figure 3A**). Differentially expressed genes were enriched and functionally annotated according to Gene Ontology (GO) and the Kyoto Encyclopedia of Genomes (KEGG). And found several enriched GO categories. GO analysis unveiled that these chemokines were notably enriched in several key biological processes (BP), including cell chemotaxis, chemokine-mediated signaling pathways, and responses to chemokines. They demonstrated enrichment in cellular components (CC), such as the external side of the plasma membrane, host cell cytoplasmic regions, and host cell cytoplasm. Moreover, molecular function (MF) analysis highlighted their involvement in chemokine activity, chemokine receptor binding, and cytokine activity. The analysis based on KEGG pathways identified several significant enrichments. From the KEGG pathway analysis, we observed that these differentially expressed chemokines were predominantly associated with pivotal pathways including viral protein interactions with cytokines and cytokine receptors, the chemokine signaling pathway, and cytokine-cytokine receptor interactions, and there is a relationship with immunity (**Figure 3B**). Moreover, Spearman correlation analysis shed light on the interplay among molecules within the chemokines family in CRC. chemokines not only regulate the process of tumorigenesis and progression through external molecules but at the same time are closely linked and interact internally revealing their close interactions and pivotal roles in the disease pathogenesis (**Figure 3C**).

### 3.3 Constructing a risk prognostic model

**Figure 4:**
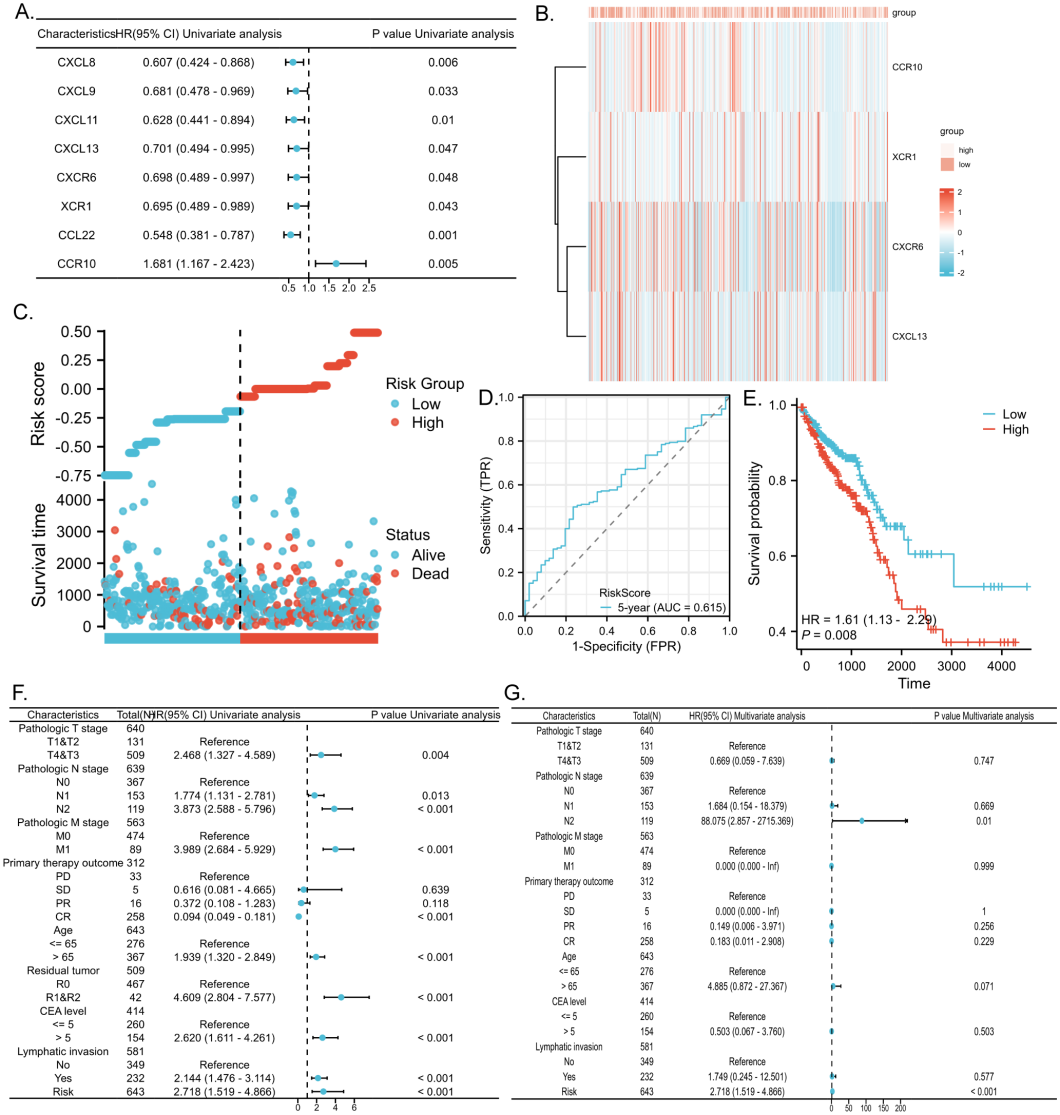
Predictive value of chemokines model for prognosis of CRC patients. **(A)** Forest plot of chemokines Cox proportional risk regression model. **(B)** Heatmap of the expression of molecules belonging to chemokines models in the TCGA database based on risk score grouping. **(C)** Distribution of risk scores based on prognostic characteristics. **(D)** Time-dependent ROC curve analysis of the AUC was used to assess the prognostic performance of the risk score for 5-year OS. **(E)** Kaplan-Meier survival curves for CRC patients grouped based on risk scores. The red line represents the high-risk group and the blue line represents the low-risk group. **(F, G)** Forest plots of univariate and multivariate Cox regression analysis of clinicopathologic characteristics with patient prognosis.

We conducted COX regression analysis on 64 chemokine molecules to elucidate the relationship between chemokine expression and CRC prognosis. The findings unveiled that 8 chemokine exhibited significant associations with survival among CRC patients (**Figure 4A**). Subsequently, a multifactorial Cox regression analysis employing expression trends consistent with four genes CXCR6, XCR1, CCR10, and CXCL13 was utilized to model prognostic risk and compute risk scores for each patient. Based on the median risk score for all patients, the 643 CRC patients were stratified into either a high-risk group (322 patients) or a low-risk group (321 patients). The combination of prognosis-related chemokine gene expression and risk grouping facilitated the construction of a heatmap illustrating survival profiles and chemokine expression in high and low-risk groups of CRC patients (**Figures 4B, C**). To assess the prognostic performance of the model, ROC analysis was conducted, affirming its efficacy in predicting CRC patient outcomes (**Figure 4D**). Furthermore, Kaplan-Meier analysis revealed that patients in the high-risk group exhibited significantly worse prognoses compared to those in the low-risk group (P<0.01) (**Figure 4E**). To comprehensively evaluate the predictive capacity of our prognostic model, univariate (**Figure 4F**, **Table 2**) and multivariate (**Figure 4G**, **Table 3**) Cox regression analyses were performed. The results underscored the utility of the risk score as an independent predictor of prognosis in patients with CRC (P<0.001).

**Table 2:** Univariate analysis of survival in CRC patients applying the TCGA dataset.

Characteristics	Total(N)	HR(95% CI) Univariate analysis	P value Univariate analysis
Pathologic T stage	640		
T1&T2	131	Reference	
T4&T3	509	2.468 (1.327 - 4.589)	0.004
Pathologic N stage	639		
N0	367	Reference	
N1	153	1.774 (1.131 - 2.781)	0.013
N2	119	3.873 (2.588 - 5.796)	< 0.001
Pathologic M stage	563		
M0	474	Reference	
M1	89	3.989 (2.684 - 5.929)	< 0.001
Primary therapy outcome	312		
PD	33	Reference	
SD	5	0.616 (0.081 - 4.665)	0.639
PR	16	0.372 (0.108 - 1.283)	0.118
CR	258	0.094 (0.049 - 0.181)	< 0.001
Age	643		
<= 65	276	Reference	
> 65	367	1.939 (1.320 - 2.849)	< 0.001
Residual tumor	509		
R0	467	Reference	
R1&R2	42	4.609 (2.804 - 7.577)	< 0.001
CEA level	414		
<= 5	260	Reference	
> 5	154	2.620 (1.611 - 4.261)	< 0.001
Lymphatic invasion	581		
No	349	Reference	
Yes	232	2.144 (1.476 - 3.114)	< 0.001
Risk	643	2.718 (1.519 - 4.866)	< 0.001

**Table 3:** Multifactorial analysis of survival in CRC patients applying the TCGA dataset.

Characteristics	Total(N)	HR(95% CI) Multivariate analysis	P value Multivariate analysis
Pathologic T stage	640		
T1&T2	131	Reference	
T4&T3	509	0.669 (0.059 - 7.639)	0.747
Pathologic N stage	639		
N0	367	Reference	
N1	153	1.684 (0.154 - 18.379)	0.669
N2	119	88.075 (2.857 - 2715.369)	0.01
Pathologic M stage	563		
M0	474	Reference	
M1	89	0.000 (0.000 - Inf)	0.999
Primary therapy outcome	312		
PD	33	Reference	
SD	5	0.000 (0.000 - Inf)	1
PR	16	0.149 (0.006 - 3.971)	0.256
CR	258	0.183 (0.011 - 2.908)	0.229
Age	643		
<= 65	276	Reference	
> 65	367	4.885 (0.872 - 27.367)	0.071
CEA level	414		
<= 5	260	Reference	
> 5	154	0.503 (0.067 - 3.760)	0.503

### 3.4 Prognostic utility of chemokines in CRC

**Figure 5:**
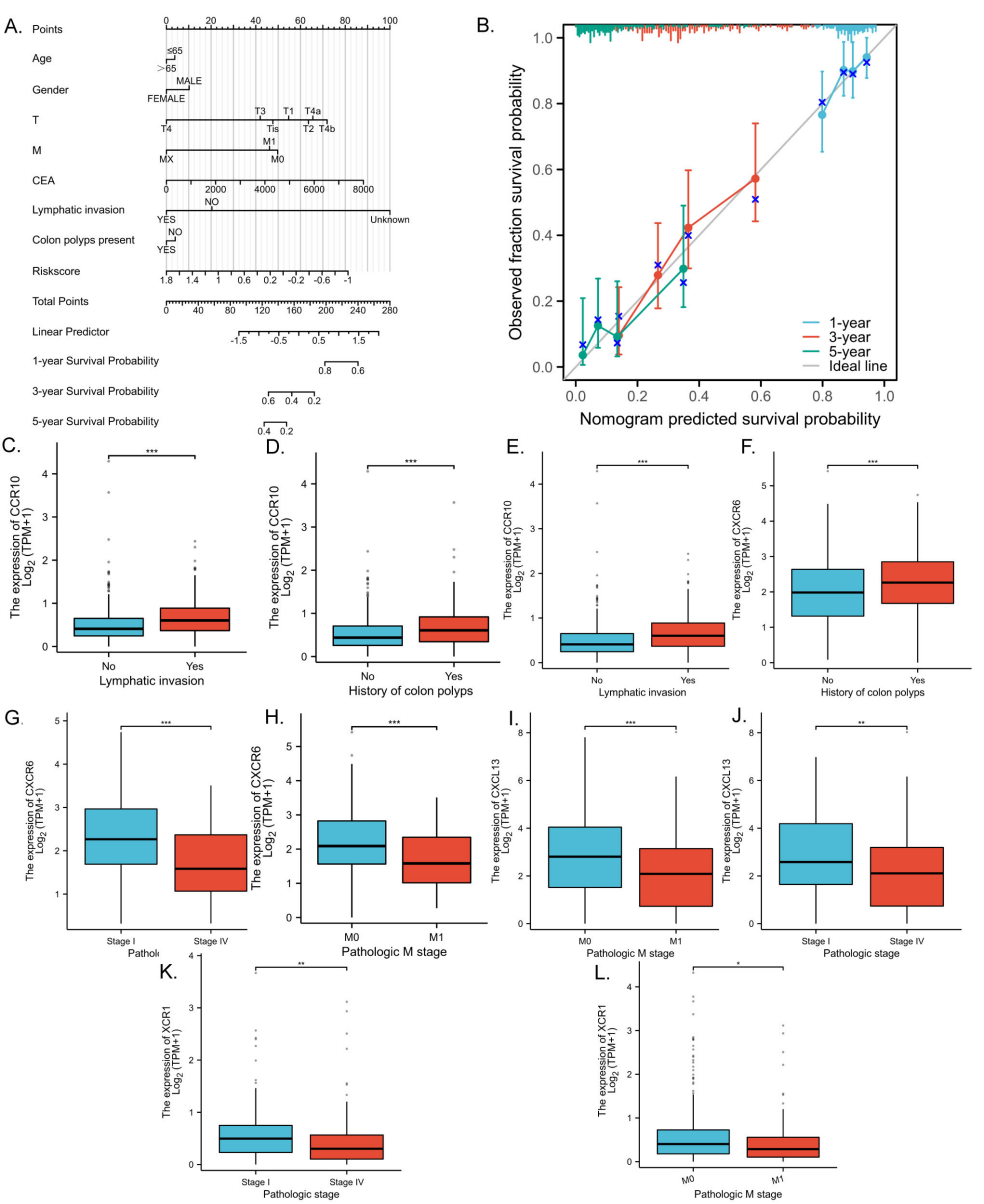
Validation of chemokines model for predicting the prognosis of CRC patients with clinical features. **(A)** Nomogram to assess the overall survival of CRC patients at 1, 3, and 5 years. **(B)** Prognostic calibration plots validate the difference between the predicted and actual probabilities of the model at different time points. **(C–L)** Statistical box plots correlating molecular expression with clinical features of CRC in the model. p < 0.05 *, p < 0.01 ** and p < 0.001 ***.

To facilitate the clinical implementation of our model and provide reliable prognostic information for individual patients, we developed a Nomogram predicting the probability of 1-, 3-, and 5-year overall survival (OS) in patients with CRC. This Nomogram was created by integrating clinical data with the risk scores generated from our prognostic model (**Figure 5A**). The prognostic Calibration plot was employed to assess the consistency between predicted and actual probabilities at various time points. Calibration statistical charts demonstrate the Nomogram plot closely mirrored the observed outcomes, indicating high prediction accuracy (**Figure 5B**). Furthermore, the expression profiles of chemokine molecules in CRC were scrutinized in conjunction with clinical features, with baseline clinical data summarized for reference (**Table 4**). Our analysis revealed distinct patterns: XCR1, CXCL13, and CXCR6 expression levels were diminished in M1-staged and advanced CRC cases, while CXCR6 expression was elevated in CRC patients with a history of polyposis, and CCR10 expression was heightened in lympho-invasive CRC. The expression of important molecules of the chemokine model is reduced in the clinical significance of increased CRC malignancy. However, CRC lymphatic infiltration may be associated with chemokine regulation, and some chemokine expression was elevated in the clinical significance of lymphatic infiltration. These findings underscore the close relationship between the molecular expression of chemokines in our model and specific clinical features (**Figures 5C–L**).

**Table 4:** Clinical data at baseline.

Characteristics	Low expression of CXCR6	High expression of CXCR6	P value
n	322	322	
Pathologic M stage, n (%)			0.003
M0	228 (40.4%)	247 (43.8%)	
M1	58 (10.3%)	31 (5.5%)	
Pathologic stage, n (%)			0.001
Stage I	43 (6.9%)	68 (10.9%)	
Stage II	110 (17.7%)	128 (20.5%)	
Stage III	99 (15.9%)	85 (13.6%)	
Stage IV	58 (9.3%)	32 (5.1%)	
Neoplasm type, n (%)			0.031
Colon adenocarcinoma	227 (35.2%)	251 (39%)	
Rectum adenocarcinoma	95 (14.8%)	71 (11%)	
Pathologic N stage, n (%)			0.005
N0	163 (25.5%)	205 (32%)	
N1	88 (13.8%)	65 (10.2%)	
N2	68 (10.6%)	51 (8%)	
Histological type, n (%)			0.008
Adenocarcinoma	285 (45%)	265 (41.9%)	
Mucinous adenocarcinoma	30 (4.7%)	53 (8.4%)	
History of colon polyps, n (%)			0.001
No	204 (36.8%)	173 (31.2%)	
Yes	70 (12.6%)	108 (19.5%)	
n	322	322	
History of colon polyps, n (%)			0.023
No	200 (36%)	177 (31.9%)	
Yes	76 (13.7%)	102 (18.4%)	
PFI event, n (%)			0.015
No	226 (35.1%)	253 (39.3%)	
Yes	96 (14.9%)	69 (10.7%)	

Characteristics	Low expression of CCR10	High expression of CCR10	P value
n	321	323	
History of colon polyps, n (%)			< 0.001
No	202 (36.4%)	175 (31.5%)	
Yes	66 (11.9%)	112 (20.2%)	
Lymphatic invasion, n (%)			< 0.001
No	203 (34.9%)	147 (25.3%)	
Yes	83 (14.3%)	149 (25.6%)	

Characteristics	Low expression of CXCL13	High expression of CXCL13	P value
n	322	322	
Pathologic M stage, n (%)			0.002
M0	226 (40.1%)	249 (44.1%)	
M1	58 (10.3%)	31 (5.5%)	
Pathologic stage, n (%)			0.010
Stage I	56 (9%)	55 (8.8%)	
Stage II	104 (16.7%)	134 (21.5%)	
Stage III	94 (15.1%)	90 (14.4%)	
Stage IV	58 (9.3%)	32 (5.1%)	
Residual tumor, n (%)			0.012
R0	224 (43.9%)	244 (47.8%)	
R1	1 (0.2%)	5 (1%)	
R2	25 (4.9%)	11 (2.2%)	
Neoplasm type, n (%)			0.004
Colon adenocarcinoma	223 (34.6%)	255 (39.6%)	
Rectum adenocarcinoma	99 (15.4%)	67 (10.4%)	

**Figure 6:**
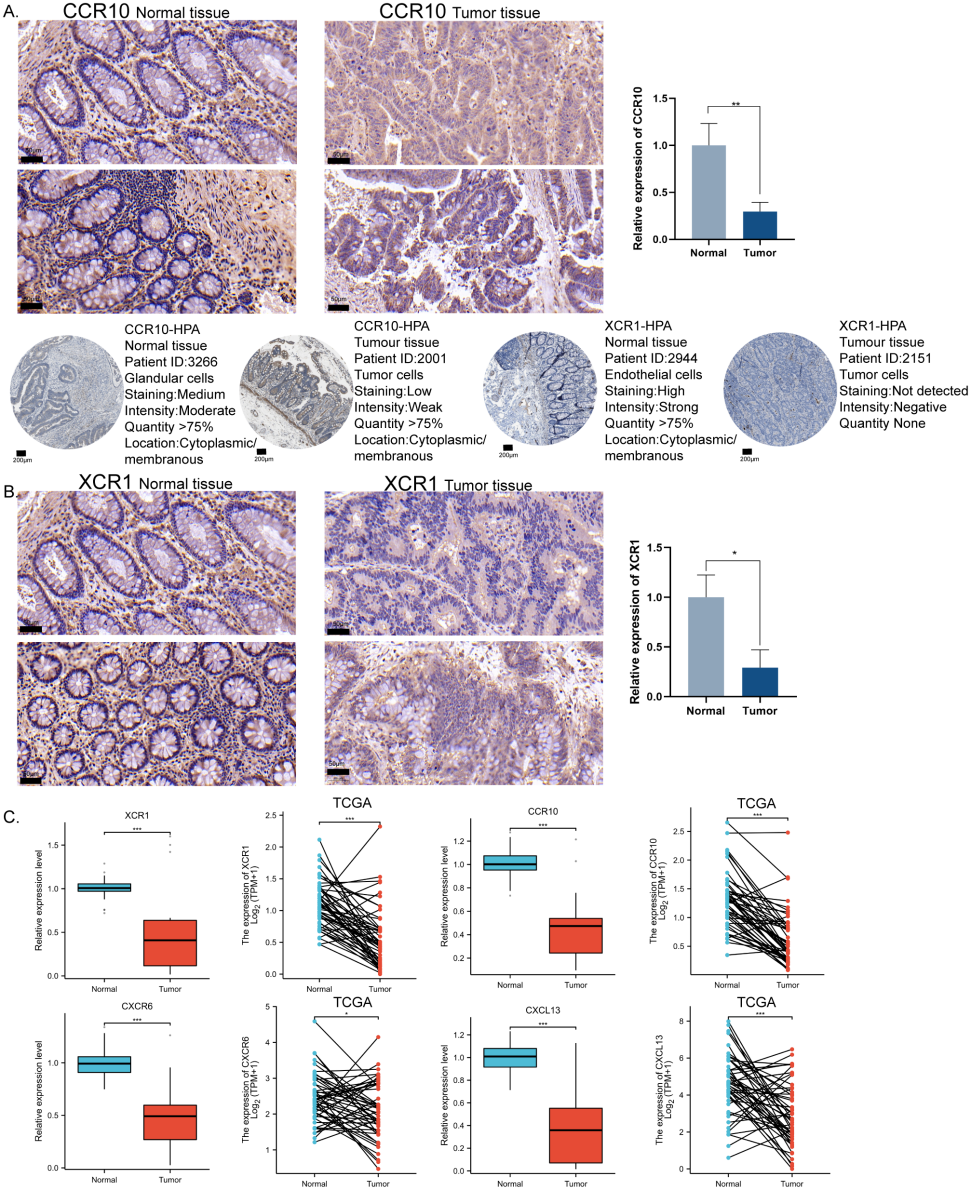
*In vitro* immunohistochemical validation of important molecules for modeling chemokines. **(A)** Representative immunohistochemical staining images showing the expression level of CCR10 in CRC tissues and normal tissues. HPA database source CCR10 expression level in CRC tissues and normal tissues. Scale bar = 50μm. **(B)** Representative immunohistochemical staining images showing the expression level of XCR1 in CRC tissues and normal tissues. HPA database source XCR1 expression level in CRC tissues and normal tissues. Scale bar = 200μm. **(C)** qRT-PCR validated the expression levels of XCR1, CCR10, CXCL13, and CXCR6 in CRC tissues versus normal tissues using TCGA paired data, two-tailed t-test, *p < 0.05, **p < 0.01, ***p < 0.001.

To validate the expression patterns observed in the CRC predictive model, the most significant expression differences in CRC, CCR10, and XCR1, were selected for protein level validation. Immunohistochemical validation of tissue samples from CRC patients was showed that mildly positive CCR10 and XCR1 expression could be observed in tumor tissues, but higher CCR10 and XCR1 expression was observed in normal tissues. In addition, we confirmed our findings by retrieving relevant information from the Human Protein Atlas (HPA) database. Immunohistochemistry results in the HPA database showed that CCR10 and XCR1 were expressed at higher and more sensitive levels in normal tissues, consistent with our experimental results (**Figures 6A, B**). QRT-PCR was used to verify the RNA expression levels of the four chemokines. Ten pairs of tumor tissues and their corresponding normal tissues were collected for RNA extraction and qRT-PCR examination. The results showed that the expression levels of the four chemokines were higher in normal tissues than in tumor tissues. TCGA-COAD paired data were also collected, and it was similarly found that at the RNA expression level, the four chemokines were expressed at higher levels in normal tissues than in tumor tissues (**Figure 6C**). Consistent with our previous observations.

### 3.5 Prognostic models are associated with immune infiltration

**Figure 7:**
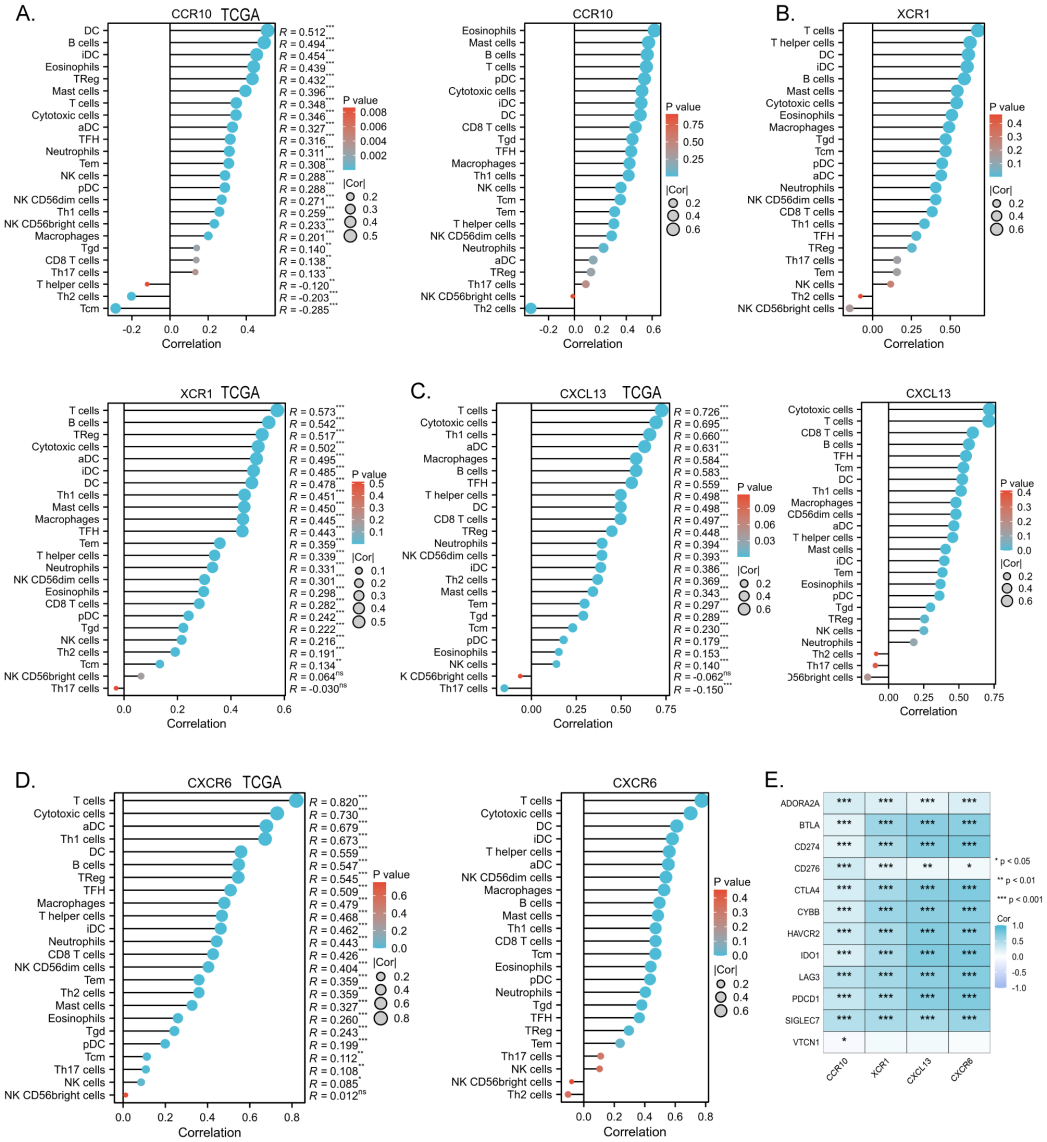
**(A)** Correlation of the chemokine model factor CCR10 with immune cell infiltration and validation of TCGA data. **(B)** Correlation of the chemokine model factor XCR1 with immune cell infiltration and validation of TCGA data. **(C)** Correlation of the chemokine model factor CXCL13 with immune cell infiltration and validation of TCGA data. **(D)** Correlation of the chemokine model factor CXCR6 with immune cell infiltration and validation of TCGA data. **(E)** Molecular correlation of chemokine model factors with common immune checkpoints, *p < 0.05, **p < 0.01, ***p < 0.001, ns means no statistical significance.

**Figure 8:**
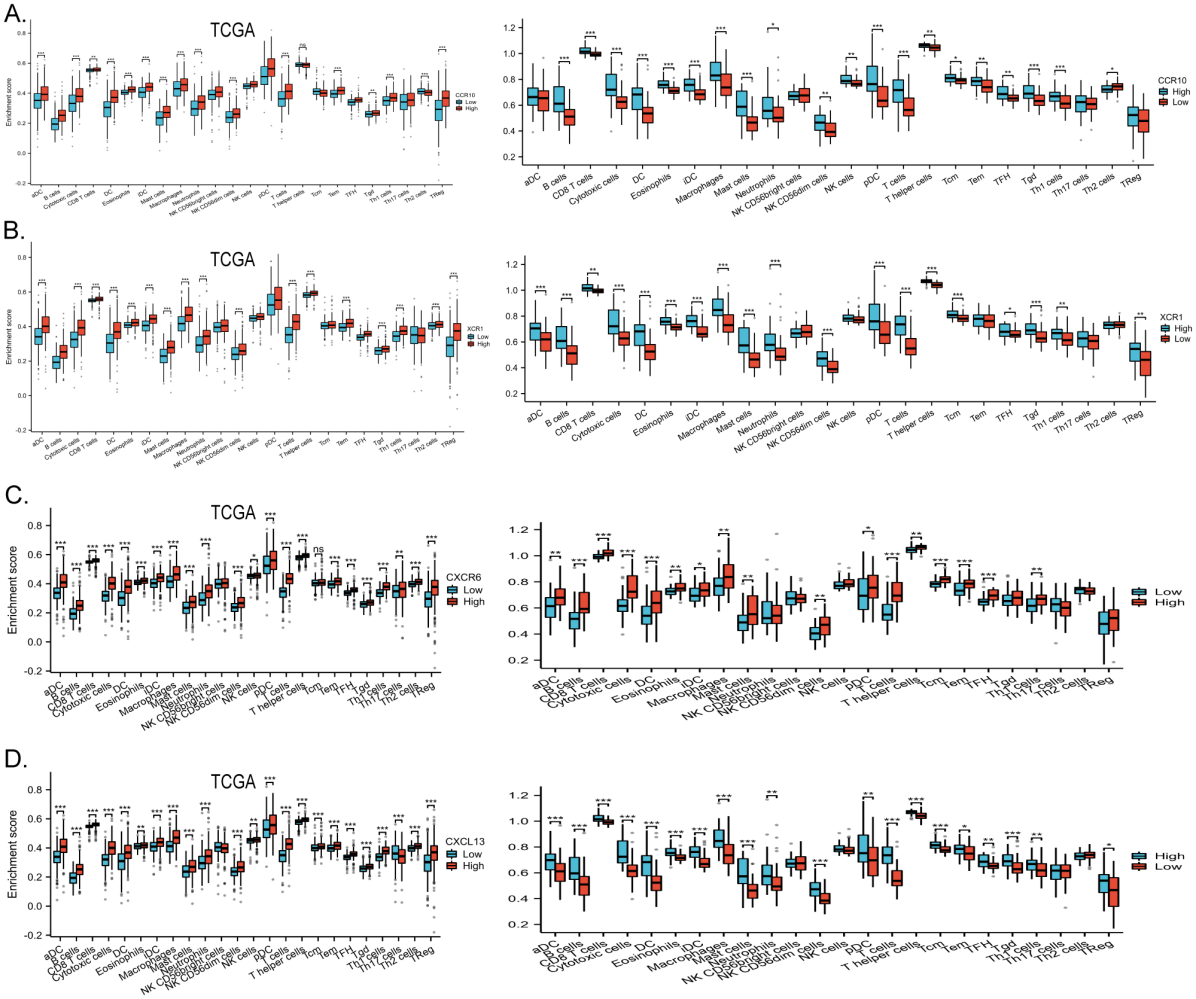
**(A)** High and low expression groups of CCR10 resulted in differences in immune cell infiltration scores, data from the clinically collected independent cohort, and TCGA data validation. **(B)** High and low expression groups of XCR1 resulted in differences in immune cell infiltration scores, data from the clinically collected independent cohort, and TCGA data validation. **(C)** High and low expression groups of CXCL13 resulted in differences in immune cell infiltration scores, data from the clinically collected independent cohort, and TCGA data validation. **(D)** High and low expression groups of CXCR6 resulted in differences in immune cell infiltration scores, data from the clinically collected independent cohort, and TCGA data validation. *p < 0.05, **p < 0.01 and ***p < 0.001.

The chemokine family plays a key role in regulating inflammatory responses and tumor immunity. Our prognostic model showed a strong association with immune infiltration. Using the single-sample gene set enrichment analysis (ssGSEA) algorithm on collected samples, we elucidated the relationship between specific molecules and immune infiltration in our prognostic model. Our results showed that CCR10 had a very strong positive correlation with both B cells and T cells. It has been documented that CCR10 interacts with CCL28 to promote plasma cell metastasis into the tumor stroma (16) (**Figure 7A**). xCR1, on the other hand, had a strong positive correlation with T cells, and DC cells (**Figure 7B**); CXCL13 was extremely correlated with both T cells and B cells, and was most strongly correlated with cytotoxic T cells all positively correlated (**Figure 7C**); CXCR6 was strongly and positively correlated with T cells, B cells and DC cells (P<0.05) (**Figure 7D**). We predicted corrections between XCR1 CCR10 CXCR6 CXCL13 and common immune checkpoint, which were strongly correlated with the expression of the clinically used immune checkpoint inhibitors PD-L1 and CTLA-4. This suggests that the present model has the potential to predict the response to immunotherapy in colorectal cancer patients (**Figure 7E**). Meanwhile, data collected from the TCGA database confirmed these results, which were consistent with our observations. Furthermore, utilizing sequencing data from our collected samples, we employed the median expression of chemokines as a delineating boundary to categorize high and low chemokine expression levels. Subsequently, we observed the infiltration of immune cells, revealing a significant correlation between the expression of chemokines and the abundance of infiltrating immune cells (P<0.05). Our analyses showed that CD8+ T cells, which are directly associated with tumor immunotherapy response, as well as T cells were more highly expressed at higher levels of all four molecules; B cells were also more highly expressed at higher levels of XCR1, CCR10, CXCL13, CXCR6. These results are consistent with the correlation analysis. These findings were validated against the TCGA database, further affirming the robustness of our results (**Figures 8A–D**). In summary, our study underscores a strong correlation between immune cell infiltration and our prognostic model, highlighting the pivotal role of chemokines in modulating the tumor microenvironment and influencing patient outcomes.

## 4 Discussion

While our comprehension of the intricate chemokine systems within tumor biology has advanced, our understanding of the multifaceted roles of chemokines and their predictive significance in various cancer forms remains somewhat limited, particularly within the scope of diverse anticancer therapies. The cornerstone of CRC management remains surgical intervention complemented by adjuvant chemotherapy and radiation therapy. Nonetheless, the adverse effects associated with radiotherapy and its suboptimal survival outcomes underscore the necessity for exploring alternative therapeutic modalities. Immunotherapy, as a burgeoning treatment paradigm, has exhibited notable progress in the realm of CRC therapeutics in recent years, outstripping radiotherapy in terms of both specificity and survival prognosis. However, a hallmark of cancer lies in tumor cells’ ability to elude immune surveillance, presenting immune evasion as a foremost obstacle to successful immunotherapy in CRC. This evasion mechanism is intricately intertwined with chemokine signaling. A considerable number of inhibitors targeting various chemokine receptors and pathways are presently under scrutiny in numerous preclinical investigations and clinical trials, yielding promising outcomes especially when employed synergistically with chemotherapy or immune checkpoint blockade. The integration of chemokine inhibitors into CRC immunotherapy regimens holds equal promise and warrants further exploration.

The interplay between tumor cells and TME is integral, underscoring the intricate relationship between TME heterogeneity and varied patient responses to therapy, thereby accentuating the pivotal role of TME in antitumor efficacy (17, 18). Chemokine receptors wield influence over the state of the tumor immune microenvironment by modulating inflammatory responses and immune cell infiltration (19, 20). Chemokines and chemokine receptors play important roles in many physiological and pathological processes and have a significant impact on the development of colorectal cancer. Chemokines are not only involved in the recruitment and activation of immune cells, but also play a key role in the proliferation, migration and invasion of tumor cells. While certain chemokines, such as serum CXCL7, have been identified as biomarkers of adverse prognosis when overexpressed in CRC(CRC), the roles of most chemokines in CRC progression remain largely unexplored (21). Chemokines in colorectal cancer may inhibit or promote tumor angiogenesis and participate in inflammatory processes. It has been found that chemokines influence tumorigenesis and metastasis by binding to vascular receptors endothelial cells through signal transduction pathways (22). CCL5 recruits fibroblasts in colorectal cancer through CCR5-SLC25A24 signaling, which increases VEGFA and transdifferentiates fibroblasts into vascular endothelial cells, promoting tumor angiogenesis and collagen synthesis, ultimately promoting tumor development (23). It has also been found that CXCL5 promotes tumor angiogenesis in a CXCR2-dependent manner both *in vitro* and *in vivo* (24). However, the role of most chemokines in CRC progression has not been reported. Another important process in tumor progression is metastasis, the migration of malignant tumor cells to areas far from the primary tumor site (25). Chemokines and their receptors may be predictors of distant metastasis in colorectal cancer. And the majority of cancer-related deaths are caused by distant metastases. Tumor cells often express specific chemokine receptors that facilitate their migration to various anatomical sites, fostering metastatic spread (26). Heightened expression of CXCR3 and CXCR4 has been implicated in promoting colon cancer metastasis to draining lymph nodes, thereby correlating with unfavorable prognoses for CRC tumors metastasizing to the liver and lungs (27). Chemokine networks have been used to treat patients with colorectal cancer and may serve as targets for drug resistance and treatment of colorectal cancer. Opportunities for therapeutic intervention by targeting chemokines and chemokine receptors such as biologics, small molecules, peptides and small interfering ribonucleic acids (siRNA), can be used to target different stages of colorectal cancer (28). It has now been shown that targeting the CCR5 receptor using Maraviroc and RNA interference can produce significant inhibition of cancer progression (29). Maraviroc in combination with chemotherapy has been enrolled in a phase 1 clinical trial (NCT01736813) and confirmed the effectiveness of CCR5 blockade. Blocking CCR5 is an effective treatment to reduce the pro-tumor inflammatory microenvironment by focusing on tumor cells and TMA (30). Additionally, the combination of Maraviroc and Pembrolizumab has been shown to improve the overall survival of colorectal cancer patients (31). To date, several CXCR4 antagonists have been developed for the treatment of these cancers (32). Current CRC treatment modalities included blocking the chemokine receptor binding process to prevent cancer development. A comprehensive investigation and molecular delineation of chemokines in CRC are therefore imperative to deepen our understanding of the antitumor immune response and prognostic determinants in CRC. Such endeavors will furnish valuable insights into therapeutic strategies aimed at mitigating tumor progression and improving patient outcomes.

More than 20 members within the CC subfamily of chemokines feature two adjacent Cysteine residues at the N-terminal end of the molecule. These chemokines exert chemotactic effects and activate various immune cell subsets, including monocytes, certain T-cell populations, B cells, eosinophils, dendritic cells (DCs), and natural killer (NK) cells, albeit they do not impact neutrophils. Studies have elucidated the ability of CCR10 to be stimulated by CCL27/CCL28, thereby facilitating the recruitment of lymphocytes (33, 34). However, in the context of CRC, there is a concomitant down-regulation in the expression of CCL27/CCL28. Therapeutic interventions aimed at enhancing the production of CCL27 have demonstrated promising anticancer properties (35, 36). The synergistic action of CCR10-CCL28 has been shown to impede invasion in oral squamous cell carcinoma, concurrently inhibiting the differentiation of osteoblastic precursor cells, thus impeding the progression of oral squamous cell carcinoma (37).

The CXC family of chemokines, ranging from CXCL1 to CXCL17, comprises proteins typically ranging between 8 to 10 kDa. These molecules exert their effects by signaling through chemokine receptors 1 to 8, thereby orchestrating the recruitment of neutrophils and lymphocytes. Both chemokines and their corresponding receptors have been implicated in either promoting or inhibiting cancer progression, potentially influencing metastasis and drug resistance in CRC, thereby serving as promising prognostic indicators (38). In a study conducted by Xianjun Yu et al., it was elucidated that CXCL13 and its receptor CXCR5 wield significant regulatory influence over the intricate tumor immune microenvironment. These molecules instigate intracellular signaling cascades within tumor cells, operating in an autocrine or paracrine manner. The CXCL13/CXCR5 axis has been identified as a key mediator of pro-tumorigenic immune responses, facilitating the recruitment of immunosuppressive cells to tumor sites. Recent research has unveiled that silencing CXCL13 effectively inhibits CRC induced by azoxymethane/glucose sodium sulfate in murine models (39). Furthermore, CXCR6 emerges as a pivotal marker for tissue-resident memory (TRM) cells across various cancer types and serves as the receptor for the chemokine CXCL16. It has been demonstrated that the anti-tumor efficacy of T cells in melanoma hinges upon the involvement of CXCR6-expressing CD8+ T cells (40). Our findings align consistently with previous research, further underscoring the applicability of our predictive model. Within the XC subfamily, prominence is bestowed upon three key chemokines: XCR1, XCL1, and XCL2. Notably, the interaction between XCR1 and its ligand XCL1 holds significant implications for organismal immune function. In a study by Qiao-Nan Guo et al., it was demonstrated that heightened XCR1 expression in clear cell renal cell carcinoma correlates with improved patient prognoses. Moreover, knockdown of XCR1 was shown to markedly enhance tumor cell proliferation and migration while dampening apoptosis (41). Our projected results suggest a potential predictive role for XCR1 in CRC(CRC), albeit further investigation is warranted to elucidate the precise mechanisms underlying XCR1’s involvement in CRC progression.

The main purpose of predictive modelling is to inform the individual about the future progression of the disease and the probability of a certain outcome, in order to guide doctors and patients in making decisions about future prevention, treatment, and rehabilitation programs (42, 43). In addition, the application of predictive models allows for the selection of appropriate and relevant patients for treatment programs. In this study, we used 64 chemokines family molecules as screening targets, narrowed down the model sample through genetic variation and difference analyses, and clarified the role of chemokine families through KEGG pathway enrichment analysis. Subsequently, four molecules strongly associated with colorectal cancer prognosis and with consistent expression trends were screened by bioinformatics analysis to form a prognostic analysis model. The actual role of the model was analyzed and fitted, and based on clinical information, it was showed that the prognostic model in this study can indeed play a predictive role and has the potential value of guiding colorectal cancer immunotherapy. Currently, in addition to traditional surgery, the use of immunotherapy and neoadjuvant chemotherapy has substantially improved the survival prognosis of patients. The main immunotherapy drug approved for CRC is anti-PD-1 monoclonal antibody, and a series of clinical trials on immunotherapy for CRC are being conducted around the world to improve the effectiveness and reduce the possibility of poor prognosis (44–46). We performed immunoassays on the model molecules, which were strongly correlated with immune checkpoint expression and closely associated with the expression of relevant immune cells. The model has value in predicting immune response in colorectal cancer patients. This study is also limited in that the patients’ use of immunotherapy or lack of the self-collected clinical data of this study, and the validation of the model is still limited. This study also has some limitations. This prediction model based on convergence factors may have some biases and statistical limitations. Firstly, the limitation of sample size in this study may impact the stability and accuracy if the model. Although we collected 88 clinical samples and integrated TCGA data, this is still a relatively small sample size compared to the world population. Additionally, the applicability and generalizability of the prediction models may vary between different populations. Second, a comprehensive analysis of all genes in a chemokine family may lead to multiple comparison problems. This could result in statistically false-positive results, although we performed an initial basic experimental validation that clarified the correlation between CRC and the predictive model. Follow-up experiment cold be helpful for validation. Meanwhile, this study is only a retrospective study based on observational data, with potential information bias and the effect of confounding variables. Although basic experimental validation was conducted, the influence of other unknown biological and environmental factors on the prediction model cannot be excluded. In summary, this study provides preliminary evidence to support the potential role of chemokines in CRC disease risk and prognosis, but further research and validation are necessary.

Our examination has culminated in the development of characteristic prognostic risk models incorporating chemokines (CCR10), CXCL13, CXCR6, and XCR1, thereby furnishing novel insights into the intricate landscape of the CRC immune microenvironment. However, it is imperative to acknowledge several limitations inherent in our study. Validation of these findings necessitates large-scale prospective investigations. Additionally, further assessment of the predictive efficacy of our model in CRC is imperative to refine prognostic classification and optimize therapeutic strategies.

## 5 Conclusion

Through a comprehensive series of exhaustive investigations, we meticulously assessed the plausible biological functions and prognostic implications of chemokines in CRC. Concurrently, we craft a novel prognostic model specifically for CRC patients. Our study represents a significant stride in elucidating the intricate role of chemokines within the context of CRC, clarifying that chemokines in CRC are extremely closely linked to immune infiltration, thereby unveiling novel potential prognostic markers and therapeutic targets.

## Data Availability

The original contributions presented in the study are included in the article/supplementary materials, further inquiries can be directed to the corresponding authors.

## References

[1] Sung H Ferlay J Siegel RL Laversanne M Soerjomataram I Jemal A. Global cancer statistics 2020: GLOBOCAN estimates of incidence and mortality worldwide for 36 cancers in 185 countries. CA: Cancer J Clin. (2021) 71:209–49. 10.3322/caac.21660 33538338

[2] Sugarbaker PH Ryan DP. Cytoreductive surgery plus hyperthermic perioperative chemotherapy to treat peritoneal metastases from colorectal cancer: standard of care or an experimental approach? Lancet Oncol. (2012) 13:e362–e9. 10.1016/S1470-2045(12)70210-3 22846841

[3] Sonnenblick A Kadouri L Appelbaum L Peretz T Sagi M Goldberg Y. Complete remission, in BRCA2 mutation carrier with metastatic pancreatic adenocarcinoma, treated with cisplatin based therapy. Cancer Biol Ther. (2011) 12:165–8. 10.4161/cbt.12.3.16292 21613821

[4] Khare T Bissonnette M Khare S. CXCL12-CXCR4/CXCR7 axis in colorectal cancer: therapeutic target in preclinical and clinical studies. Int J Mol Sci. (2021) 22:7371. 34298991 10.3390/ijms22147371 PMC8305488

[5] Yue Y Zhang Q Sun Z. CX3CR1 acts as a protective biomarker in the tumor microenvironment of colorectal cancer. Front Immunol. (2022) 12:758040. 35140706 10.3389/fimmu.2021.758040 PMC8818863

[6] Wang D Yang L Yu W Wu Q Lian J Li F. Colorectal cancer cell-derived CCL20 recruits regulatory T cells to promote chemoresistance via FOXO1/CEBPB/NF-κB signaling. J immunotherapy cancer. (2019) 7:1–15. 10.1186/s40425-019-0701-2 PMC6688336 31395078

[7] Zlotnik A Yoshie O. The chemokine superfamily revisited. Immunity. (2012) 36:705–16. 10.1016/j.immuni.2012.05.008 PMC3396424 22633458

[8] Mollica Poeta V Massara M Capucetti A Bonecchi R. Chemokines and chemokine receptors: new targets for cancer immunotherapy. Front Immunol. (2019) 10:379. 30894861 10.3389/fimmu.2019.00379 PMC6414456

[9] Choi J Selmi C Leung PS Kenny TP Roskams T Gershwin ME. Chemokine and chemokine receptors in autoimmunity: the case of primary biliary cholangitis. Expert Rev Clin Immunol. (2016) 12:661–72. 10.1586/1744666X.2016.1147956 PMC4935758 26821815

[10] Nagarsheth N Wicha MS Zou W. Chemokines in the cancer microenvironment and their relevance in cancer immunotherapy. Nat Rev Immunol. (2017) 17:559–72. 10.1038/nri.2017.49 PMC5731833 28555670

[11] Cao Y Jiao N Sun T Ma Y Zhang X Chen H. CXCL11 correlates with antitumor immunity and an improved prognosis in colon cancer. Front Cell Dev Biol. (2021) 9:646252. 33777950 10.3389/fcell.2021.646252 PMC7991085

[12] Fuji S Utsunomiya A Inoue Y Miyagi T Owatari S Sawayama Y. Outcomes of patients with relapsed aggressive adult T-cell leukemia-lymphoma: clinical effectiveness of anti-CCR4 antibody and allogeneic hematopoietic stem cell transplantation. Haematologica. (2018) 103:e211. 29371324 10.3324/haematol.2017.184564 PMC5927966

[13] Micallef IN Stiff PJ Nademanee AP Maziarz RT Horwitz ME Stadtmauer EA. Plerixafor plus granulocyte colony-stimulating factor for patients with non-Hodgkin lymphoma and multiple myeloma: long-term follow-up report. Biol Blood Marrow Transplantation. (2018) 24:1187–95. 10.1016/j.bbmt.2018.01.039 PMC6091693 29410180

[14] Franceschini A Szklarczyk D Frankild S Kuhn M Simonovic M Roth A. STRING v9. 1: protein-protein interaction networks, with increased coverage and integration. Nucleic Acids Res. (2012) 41:D808–D15. 10.1093/nar/gks1094 PMC3531103 23203871

[15] Hughes CE Nibbs RJ. A guide to chemokines and their receptors. FEBS J. (2018) 285:2944–71. 10.1111/febs.14466 PMC6120486 29637711

[16] Xia J Xie Z Niu G Lu Z Wang Z Xing Y. Single-cell landscape and clinical outcomes of infiltrating B cells in colorectal cancer. Immunology. (2023) 168:135–51. 10.1111/imm.13568 36082430

[17] Mantovani A Allavena P Sica A Balkwill F. Cancer-related inflammation. nature. (2008) 454:436–44. 10.1038/nature07205 18650914

[18] Colotta F Allavena P Sica A Garlanda C Mantovani A. Cancer-related inflammation, the seventh hallmark of cancer: links to genetic instability. Carcinogenesis. (2009) 30:1073–81. 10.1093/carcin/bgp127 19468060

[19] Griffith JW Sokol CL Luster AD. Chemokines and chemokine receptors: positioning cells for host defense and immunity. Annu Rev Immunol. (2014) 32:659–702. 24655300 10.1146/annurev-immunol-032713-120145

[20] Pączek S Łukaszewicz-Zając M Mroczko B. Chemokines—what is their role in colorectal cancer? Cancer Control. (2020) 27:1073274820903384. 32103675 10.1177/1073274820903384 PMC7066593

[21] Li L Zhang L Zhang T Qi X Cheng G Xia L. Serum chemokine CXCL7 as a potential novel biomarker for obstructive colorectal cancer. Front Oncol. (2021) 10:599363. 33643903 10.3389/fonc.2020.599363 PMC7902867

[22] Solimando AG Summa SD Vacca A Ribatti D. Cancer-associated angiogenesis: the endothelial cell as a checkpoint for immunological patrolling. Cancers. (2020) 12:3380. 33203154 10.3390/cancers12113380 PMC7696032

[23] Gao L-F Zhong Y Long T Wang X Zhu J-X Wang X-Y. Tumor bud-derived CCL5 recruits fibroblasts and promotes colorectal cancer progression via CCR5-SLC25A24 signaling. J Exp Clin Cancer Res. (2022) 41:81. 35241150 10.1186/s13046-022-02300-w PMC8892738

[24] Chen C Xu Z-Q Zong Y-P Ou B-C Shen X-H Feng H. CXCL5 induces tumor angiogenesis via enhancing the expression of FOXD1 mediated by the AKT/NF-κB pathway in colorectal cancer. Cell Death disease. (2019) 10:178. 30792394 10.1038/s41419-019-1431-6 PMC6385313

[25] Chambers AF Groom AC MacDonald IC. Dissemination and growth of cancer cells in metastatic sites. Nat Rev Cancer. (2002) 2:563–72. 10.1038/nrc865 12154349

[26] Vicari AP Caux C. Chemokines in cancer. Cytokine Growth factor Rev. (2002) 13:143–54. 10.1016/s1359-6101(01)00033-8 11900990

[27] Murakami T Kawada K Iwamoto M Akagami M Hida K Nakanishi Y. The role of CXCR3 and CXCR4 in colorectal cancer metastasis. Int J cancer. (2013) 132:276–87. 10.1002/ijc.27670 22689289

[28] Zacarías NVO Bemelmans MP Handel TM de Visser KE Heitman LH. Anticancer opportunities at every stage of chemokine function. Trends Pharmacol Sci. (2021) 42:912–28. 10.1016/j.tips.2021.08.001 34521537

[29] Pervaiz A Zepp M Georges R Bergmann F Mahmood S Faiza S. Antineoplastic effects of targeting CCR5 and its therapeutic potential for colorectal cancer liver metastasis. J Cancer Res Clin Oncol. (2021) 147:73–91. 32902795 10.1007/s00432-020-03382-9 PMC7810651

[30] Halama N Zoernig I Berthel A Kahlert C Klupp F Suarez-Carmona M. Tumoral immune cell exploitation in colorectal cancer metastases can be targeted effectively by anti-CCR5 therapy in cancer patients. Cancer Cell. (2016) 29:587–601. 27070705 10.1016/j.ccell.2016.03.005

[31] Haag GM Halama N Springfeld C Grün B Apostolidis L Zschaebitz S. Combined PD-1 inhibition (Pembrolizumab) and CCR5 inhibition (Maraviroc) for the treatment of refractory microsatellite stable (MSS) metastatic colorectal cancer (mCRC): First results of the PICCASSO phase I trial. J Clin Oncol. (2020) 38.

[32] Tahirovic YA Pelly S Jecs E Miller EJ Sharma SK Liotta DC. Small molecule and peptide-based CXCR4 modulators as therapeutic agents. A patent review for the period from 2010 to 2018. Expert Opin Ther patents. (2020) 30:87–101. 10.1080/13543776.2020.1707186 31854208

[33] Wang W Soto H Oldham ER Buchanan ME Homey B Catron D. Identification of a novel chemokine (CCL28), which binds CCR10 (GPR2). J Biol Chem. (2000) 275:22313–23. 10.1074/jbc.M001461200 10781587

[34] Jarmin DI Rits M Bota D Gerard NP Graham GJ Clark-Lewis I. Cutting edge: identification of the orphan receptor G-protein-coupled receptor 2 as CCR10, a specific receptor for the chemokine ESkine. J Immunol. (2000) 164:3460–4. 10.4049/jimmunol.164.7.3460 10725696

[35] Dimberg J Hugander A Wågsäter D. Protein expression of the chemokine, CCL28, in human colorectal cancer. Int J Oncol. (2006) 28:315–9. 16391784

[36] Gao J Kanagawa N Motomura Y Yanagawa T Sugita T Hatanaka Y. Cotransduction of CCL27 gene can improve the efficacy and safety of IL-12 gene therapy for cancer. Gene Ther. (2007) 14:491–502. 17203106 10.1038/sj.gt.3302892

[37] Park J Zhang X Lee SK Song N-Y Son SH Kim KR. CCL28-induced RARβ expression inhibits oral squamous cell carcinoma bone invasion. J Clin Invest. (2019) 129:5381–99. 10.1172/JCI125336 PMC6877303 31487270

[38] Cabrero-de Las Heras S Martínez-Balibrea E. CXC family of chemokines as prognostic or predictive biomarkers and possible drug targets in colorectal cancer. World J gastroenterology. (2018) 24:4738. 10.3748/wjg.v24.i42.4738 PMC6235799 30479461

[39] Zhao Q Guo J Wang G Bi Y Cheng X Liao Y. CXCL13 promotes intestinal tumorigenesis through the activation of epithelial AKT signaling. Cancer Letters. (2021) 511:1–14. 33894331 10.1016/j.canlet.2021.04.012

[40] Di Pilato M Kfuri-Rubens R Pruessmann JN Ozga AJ Messemaker M Cadilha BL. CXCR6 positions cytotoxic T cells to receive critical survival signals in the tumor microenvironment. Cell. (2021) 184:4512–30. e22. 34343496 10.1016/j.cell.2021.07.015 PMC8719451

[41] Chang X Cao Y Fu WL Tang XF Wang YL Lv YF. Overexpression of chemokine receptor lymphotactin receptor 1 has prognostic value in clear cell renal cell carcinoma. Mol Genet Genomic Med. (2021) 9:e1551. 33377624 10.1002/mgg3.1551 PMC7963425

[42] Tripepi G Jager KJ Dekker FW Zoccali C. Statistical methods for the assessment of prognostic biomarkers (Part I): discrimination. Nephrol Dialysis transplantation. (2010) 25:1399–401. 10.1093/ndt/gfq018 20139066

[43] Tripepi G Jager KJ Dekker FW Zoccali C. Statistical methods for the assessment of prognostic biomarkers (part II): calibration and re-classification. Nephrol Dialysis Transplantation. (2010) 25:1402–5. 10.1093/ndt/gfq046 20167948

[44] Chalabi M Fanchi LF Dijkstra KK Van den Berg JG Aalbers AG Sikorska K. Neoadjuvant immunotherapy leads to pathological responses in MMR-proficient and MMR-deficient early-stage colon cancers. Nat Med. (2020) 26:566–76. 10.1038/s41591-020-0805-8 32251400

[45] Le DT Kim TW Van Cutsem E Geva R Jäger D Hara H. Phase II open-label study of pembrolizumab in treatment-refractory, microsatellite instability–high/mismatch repair–deficient metastatic colorectal cancer: KEYNOTE-164. J Clin Oncol. (2020) 38:11–9. 10.1200/JCO.19.02107 PMC7031958 31725351

[46] Zhang X Wu T Cai X Dong J Xia C Zhou Y. Neoadjuvant immunotherapy for MSI-H/dMMR locally advanced colorectal cancer: new strategies and unveiled opportunities. Front Immunol. (2022) 13:795972. 35371084 10.3389/fimmu.2022.795972 PMC8968082

